# Severe vision impairment and blindness in hospitalized patients: a retrospective nationwide study

**DOI:** 10.1186/s12886-021-02021-2

**Published:** 2021-06-22

**Authors:** Ché Matthew Harris, Scott Mitchell Wright

**Affiliations:** Department of Medicine, Johns Hopkins School of Medicine, Johns Hopkins Bayview Medical Center, Baltimore, Maryland United States of America

## Abstract

**Background:**

Outcomes among hospitalized patients with severe vision impairment or blindness have not been extensively explored. This study sought to determine clinical and resource utilization outcomes in patients with severe vision impairment/blindness (SVI/B). Because obesity is very common among those who are hospitalized, we also sought to understand its impact among patients with SVI/B.

**Methods:**

We conducted a retrospective study using the National Inpatient Sample for the year 2017; hospitalized adults with and without SVI/B were compared. In addition, for all patients with SVI/B, we compared those with and without obesity. Multiple logistic regression and linear analysis were used to evaluate mortality, disposition, length of stay, and hospital charges; the analyses were adjusted for multiple variables including age, sex, and race.

**Results:**

30,420,907 adults were hospitalized, of whom 37,200 had SVI/B. Patients with SVI/B were older (mean age ± SEM: 66.4 ± 0.24 vs. 57.9 ± 0.09 years, *p* < 0.01), less likely to be female (50 % vs. 57.7 %, *p* < 0.01), more frequently insured by Medicare (75.7 % vs. 49.2 %, *p* < 0.01), and had more comorbidities (Charlson comorbidity score ≥ 3: 53.2 % vs. 27.8 %, *p* < 0.01). Patients with SVI/B had a higher in-hospital mortality rate (3.9 % vs. 2.2 %; *p* < 0.01), and had lower odds to be discharged home after hospital discharge (adjusted Odds Ratio {aOR} =0.54, [Confidence Interval (CI) 0.51–0.58]; *p* < 0.01) compared to those without SVI/B. Hospital charges were not significantly different (adjusted Mean Difference {aMD} = $247 CI [-$2,474-2,929]; *p* = 0.85) but length of stay was longer (aMD = 0.5 days CI [0.3–0.7]; *p* < 0.01) for those with SVI/B. Patients with vision impariment who were also obese had higher total hospital charges compared to those without obesity (mean difference: $9,821 [CI $1,375-$18,268]; *p* = 0.02).

**Conclusions:**

Patients admitted to American hospitals in 2017 who had SVI/B had worse clinical outcomes and greater resources utilization than those without SVI/B. Hospital-based healthcare providers who understand that those with SVI/B may be at risk for worse outcomes may be optimally positioned to help them to receive the best possible care.

**Supplementary Information:**

The online version contains supplementary material available at 10.1186/s12886-021-02021-2.

## Background

Over 3 million adults in the United States are visually impaired or blind, and up to 80 million have eye diseases that may ultimately lead to blindness [1]. Furthermore, the annual economic impact of blindness is estimated to be over 35 billion dollars [2]. As the population ages, the number of Americans with blindness is expected to double by the year 2030 [1]. As such, an increasing number of patients with blindness will be hospitalized; hospital providers of all disciplines will invariably become progressively more involved in their care. Unfortunately, few studies have sought to screen for and consider poor vision during hospitalizations when visual disturbances are not related to the chief reason for admission [3]. While it has been established that vision impairment is associated with inpatient complications outcomes, such as falls and delirium [4, 5], no US based national study has investigated in-hospital mortality in patients with severe vision impairment or blindness (SVI/B). Morse determined that older Medicare recipients with severe vision loss utilized more healthcare resources compared to those without this disability [6]. However, the impact of SVI/B on a broader age range of patient has not been studied on a national level. We used the National Inpatient Sample (NIS) database for the year 2017, and hypothesized that hospitalized patients with SVI/B would have higher in-hospital mortality, less likely to be discharged home following hospitalization, have longer hospital stays, and greater hospital charges compared to those without SVI/B.

Given the worsening obesity pandemic with worldwide prevalence tripling from 1975 to 2016 [7] (and continuing to increase in prevalence in the United States from 39 % to 2016 to 42 % in 2017 [8, 9]) and the uncertain effect it was having on patients admitted who had SVI/B, a subgroup analysis was also carried out to investigate the additive impact of obesity on these same outcome variables. In fact, research has shown positive associations between obesity and development of debilitating eye conditions such as cataracts, retinal vein occlusion, and age-related macular degeneration [10]. Thus, determining overall SVI/B prevalence and outcomes in hospitalized patients may better highlight its pervasiveness and ultimate deleterious consequences. This in turn may increase obesity eye disease awareness and possibly even obesity interventional strategies initiated or continued among ophthalmologists (such as weight loss counsel, weight loss referrals and patient education on vision loss as consequence of obesity).

## Methods

### Setting / database

 This study used the 2017 NIS database, available through the Agency for Healthcare Research and Quality provided by the Healthcare Cost and Utilization Project [11]. NIS is the largest United States based publicly available all-payer inpatient health care database [11]. It approximates a 20 % stratified sample of US hospital discharges from 46 participating states. The NIS has data for more than 7 million unweighted hospital stays per year. When weighted to represent all admissions, it estimates more than 35 million hospitalizations annually, and represents about 95 % of US hospitalizations. Strata include hospital size/volume, teaching status, geographic region, and hospital ownership. Data from 2017 NIS uses the International Classification of Diseases, 10th Revision, Clinical Modification (ICD-10-CM) coding system for all discharge diagnoses.

### Study population, patient and hospital characteristics, and o utcomes

All patients ≥ 18 years of age were included in the sample. We then identified selected patients with bilateral severe visual impairment or bilateral blindness as described by their ICD-10 CM codes (eye category 2 through 5 for either eye): https://www.icd10monitor.com/looking-at-new-icd-10-cm-codes-for-blindness (updated September 27th 2017). In addition, ICD-10 code H54.0 was also used for bilateral blindness. Supplementary Table A with ICD-10 codes provides specific descriptions of categories for each level of SVI/B. Severe vision impairment has been defined as individuals with visual acuity worse than 6/60, and blindness as those with visual acuity worse than 3/60 [12].

ICD-10 codes used for our subgroup analysis to study the impact of obesity as a secondary diagnosis on patients with SVL/B was also retrieved (See Supplementary A table for ICD-10 codes and corresponding diagnoses). For adults, obesity is defined as having a Body Mass Index (BMI) of 30 or greater [13]. BMI is calculated by taking the individual’s weight in kilograms and dividing it by their height in meters squared.

Data was collected and adjusted for select hospital and patient characteristics including hospital bed size (capacity), hospital teaching status (teaching or non-teaching), hospital geographic region (Northeastern, Midwestern, Southern and Western regions), age, gender (male and female only), race (White, Black, Hispanic, Asian or Pacific Islander), insurance (Medicare, Medicaid, Private Insurance, Uninsured), median household income (1. $1-$38,999 2. $39,000-$47,999 3. $48,000-$62,999 4. $63,000 or more), based on home zip code, and the Charlson comorbidity index (CCI: score 0 = no comorbidities score 1 = low comorbidity burden, score 2 = moderate comorbidity burden, and score 3 or greater = high comorbidity burden). The CCI has been used extensively in clinical research; it is commonly used to assess mortality risk and it is supported by extensive validity evidence [14]. Higher scores have been associated with mortality or greater healthcare resource use [15].

The primary clinical outcome was mortality during hospitalization; secondary outcomes were total hospital charges which represent the amount a hospital bills for each individual case [16], length of stay (LOS), and disposition after hospitalization. Disposition indicates the discharge location or where patients go after hospitalization. This is most often home, but not infrequently can be elsewhere including venues such as other hospitals, inpatient hospice, inpatient rehabilitation facilities, and nursing homes [17].

 Our Institutional Review Board designated this work as being exempt from detailed review (IRB review number: 00257552).

### Statistical analyses

Comparisons were examined between patients with and without SVI/B using Pearson’s χ^2^ tests and one-way analysis of variance to test categorical and continuous variables. Analyses were also carried out within the SVI/B patient cohort assessing those with and without obesity. The primary and secondary outcomes were adjusted for all of the patient demographics and hospital characteristics shown in Table 1, as well as the CCI and select specific comorbidities described in Table 2.

 Adjusted odds ratios [aOR] and adjusted mean differences [aMD] from multivariate logistic and linear regression analyses were obtained. Binary outcomes under logistic regression analyses (in-hospital mortality and discharge disposition) were studied. Linear regression was used to study continuous outcome variables (including total hospital charges and LOS). Stata 15.0 statistical software (Stata Corp, College Station, TX) was used and permitted us to account for design complexity (stratification, weighting, and clustering) [18]. The p-values for this study were 2 sided and type I error significance level was set at 0.05.

## Results

In 2017, 35,769,613 adults ≥ 18 years of age were hospitalized in the United States. From this group, 37,200 patients were severely visually impaired or blind. Demographic data is shown in Table 1 and compares patients with and without SVI/B. Patients with SVI/B were older (mean age ± SEM: 66.4 ± 0.24 vs. 57.9 ± 0.09 years, *p* < 0.01), less likely to be female (50 % vs. 57.7 %, *p* < 0.01), and a higher proportion were insured by Medicare (75.7 % vs. 49.2 %, *p* < 0.01). Table 2 displays that patients with SVI/B had a greater comorbidity burden (Charlson comorbidity score ≥ 3: 53.2 % vs. 27.8 %, *p* < 0.01), as well as higher rates of vascular and pulmonary comorbidities.

**Table 1:** Patient and Hospital Demographics: Patients with and without severe visual impairment/Blindness (SVI/B) from the National Inpatient Sample Database (2017)*

	Patients without SVI/B	Patients with SVI/B	p-value
**Total**	30,363,917 (99.8)	37,200 (0.2)	
**Age in years**, mean $$\pm$$ SE	57.9 $$\pm$$ 0.09	66.4 $$\pm$$ 0.24	< 0.01
**Female**, n (%)	17,519,980 (57.7)	18,600 (50.0)	< 0.01
**Race**, n (%)			< 0.01
White	20,404,552 (67.2)	20,832 (56.0)	
Black	4,615,315 (15.2)	9,114 (24.5)	
Hispanic	3,340,030 (11.0)	5,022 (13.5)	
Asian or Pacific Islander	819,825 (2.7)	818 (2.2)	
**Insurance**, n (%)			< 0.01
Medicare	14,939,047 (49.2)	28,160 (75.7)	
Medicaid	5,799,508 (19.1)	5,056 (14.8)	
Private	8,350,077 (27.5)	3,013(8.1)	
Uninsured	1,214,556 (4.0)	483 (1.3)	
**Median income (USD)**, n (%)			< 0.01
$1-$38,999	9,230,630 (30.4)	14,024 (37.7)	
$39,000-$47,999	8,046,438 (26.5)	9,672 (26.0)	
$48,000-$62,999	7,105,156 (23.4)	7,551 (20.3)	
$63,000 or more	5,920,963 (19.5)	5,877 (15.8)	
**Hospital Bed size, n (%)**			0.14
Small	6,103,147 (20.1)	7,068 (19.0)	
Medium	8,957,355 (29.5)	10,899 (29.3)	
Large	15,273,050 (50.3)	19,158 (51.5)	
**Hospital Region, n (%)**			< 0.01
Northeast	5,678,052 (18.7)	5,840 (15.7)	
Midwest	6,801,517 (22.4)	7,886 (21.2)	
South	11,933,019 (39.3)	15,438 (41.5)	
West	5,890,599 (19.4)	7,960 (21.4)	
**Teaching hospital, n (%)**			0.17
Non-teaching, n (%)	3,643,670 (12.0)	4,203 (11.3)	
Teaching, n (%)	26720,247 (88.0)	32,996 (88.7)	

*Analyses used Pearson’s χ^2^ test and one-way analysis of variance for categorical and continuous variables respectively.

SVI/B: Severe Vision Impairment/Blindness

**Table 2:** Associated Co-morbidities of Patients with and without Severe Vision Impairment/Blindness (SVI/B)

Co-morbidities	Patients without SVI/B N (%)	Patients with SVI/B N (%)	p-value
**Total**	30,363,917 (99.8)	37,200 (0.2)	
**Charlson comorbidity score**			< 0.01
0	11,902,655 (39.2)	5,691 (15.3)	
1	5,860,235 (19.3)	5,914 (15.9)	
2	4,099,128 (13.5)	5,580 (15.0)	
3 or more	8,441,168 (27.8)	19,790 (53.2)	
Opioid use	637,642 (2.1)	446 (1.2)	< 0.01
Tobacco use	5,222,593 (17.2)	3,720 (10.2)	< 0.01
Alcohol use	1,366,376 (4.5)	706 (1.9)	< 0.01
Depression	242,911 (0.8)	334 (0.9)	0.68
Chronic lung disease	4,736,771 (15.6)	5,914 (15.9)	< 0.01
Hypertension	15,880,328 (52.3)	26,226 (70.5)	< 0.01
Diabetes with complications*	4,433,131 (14.6)	14,619 (39.3)	< 0.01
Peripheral vascular disease	1,457,468 (4.8)	3,980 (10.7)	< 0.01

SVI/B: Severe Vision Impairment/Blindness

*Diabetes with complications. Complications include but not limited to retinopathy, neuropathy, nephropathy, foot ulcers (as described: https://www.icd10data.com/ICD10CM/Index/D/Diabetes%2c_diabetic#31090)

### Patient clinical and resource utilization outcomes

Table 3 shows adult patients with SVI/B had higher rates of mortality compared to those without SVI/B (3.9 % vs. 2.2 %; *p* < 0.01). This finding held after adjusting for potential confounders where in-hospital mortality for patients with SVI/B remained higher compared to those without SVI/B (adjusted Odds Ratio (aOR) = 1.2, [Confidence Interval (CI) 1.0-1.4]; *p* = 0.01). Patients with SVI/B had a lower odds to be discharged to home after hospital discharge (adjusted Odds Ratio {aOR} =0.54, [Confidence Interval (CI) 0.51–0.58]; *p* < 0.01). Total hospital charges were not significantly different (adjusted Mean Difference {aMD} = $247 CI [-$2,474-2,929]; *p* = 0.85) between groups, but LOS was longer (aMD = 0.5 days CI [0.3–0.7]; *p* < 0.01) for those with SVI/B.

**Table 3:** Odd ratios and differences for in-hospital outcomes in patients with and without severe vision impairment/blindness (SVI/B) ages 18 years and older from the National Inpatient Sample (2017)

Outcome	Patients without SVI/B *N* = 30,363,917	Patients with SVI/B *N* = 37,200	Univariate Odds Ratio	(95 % CI)	P-value	Multivariate Odds Ratio	(95 % CI)	P-value
In-hospital mortality, n (%)	668,008 (2.2)	1,450 (3.9)	1.8	(1.6-2.0)	< 0.01	1.2	(1.0-1.4)	0.01
Discharged to home, n (%)	19,250,723 (63.4)	14,656 (39.4)	0.37	(0.35–0.39)	< 0.01	0.54	(0.51–0.58)	< 0.01
			**Univariate** **Mean Difference**			**Multivariate** **Mean Difference**		
Mean length of stay, days	4.72	6.05	1.33	(1.18–1.48)	< 0.01	0.5	(0.3–0.7)	< 0.01
Mean charge per case, US dollars	53,388	59,900	$6,512	(4,211-8,811)	< 0.01	$247	(-2474–2929)	0.85

*Variables adjusted for confounders in multivariate analysis include age, gender, race, median household income, insurance and comorbidities measured using the Charlson comorbidity index), hospital bed size, teaching status, urban location, and region

SVI/B: Severe vision impairment/blindness

### Subgroup analysis focused on obesity

Among patients with SVI/B, 32,201 (86.5 %) were not obese and 4,999 (13.5 %) were classified as obese. Patients with obesity were younger (mean age ± SEM: 61.5 ± 0.53 vs. 67.2 ± 0.27 years, *p* < 0.01) and a higher proportion were female (58.8 % vs. 48.6 %, *p* < 0.01). Patients with obesity had higher comorbidities (Charlson comorbidity scores ≥ 3: 65.0 % vs. 51.8 %, *p* < 0.01). Table 4 shows that obese status was not associated with an altered LOS, odds of mortality, or likelihood of being discharged to home following the hospitalization. However, SVI/B patients with obesity had higher total hospital charges compared to those without obesity (mean difference: $9,821 [CI $1,375-$18,268]; *p* = 0.02).

**Table 4:** Differences and odds ratios for in-hospital outcomes in SVI/B patients with and without obesity ages 18 years and older from the National Inpatient Sample (2017)

Outcome	Non-obese and SVI/B *N* = 32,201	Obese and SVI/B *N *= 4,999	Univariate Mean Difference	(95 % CI)	P-value	Multivariate Mean Difference	(95 % CI)	P-value	
Mean length of stay, days	6.0	6.4	0.4	(-0.3-1.0)	0.06	0.5	(-0.1- 1.2)	0.12	
Mean charge per case, US dollars	$58,882	$67,456	$8,574	(1,805 − 15,662)	0.01	$9,821	(1,375 − 18,268)	0.02	
			**Univariate** **Odds Ratio**			**Multivariate** **Odds Ratio**			
In-hospital mortality, n (%)	1,320 (4.1)	154 (3.1)	0.7	(0.5-1.0)	0.1	0.64	(0.4-1.0)	0.07	
Discharged to home, n (%)	12,268 (38.1)	2,154 (43.1)	1.2	(1.0-1.3)	< 0.01	1.0	(0.9–1.2)	0.68	

*Variables adjusted for confounders in multivariate analysis include age, gender, race, median household income, insurance and comorbidities measured using the Charlson comorbidity index), hospital bed size, teaching status, urban location, and region

SVI/B: Severe vision impairment/blindness

## Discussion

Millions of Americans have vision impairment or are blind [1, 19]. As rates continue to climb with the aging of the population [1], inpatient providers will encounter more patients with SVI/B and they will be expected to effectively manage this vulnerable population. This study shows that compared to those without SVI/B, patients with SVI/B who are hospitalized have higher mortality rates, longer LOS, and are more likely to be discharged to sites other than home. Moreover, among patients with SVI/B, those who were obese had higher total hospital charges than their non-obese counterparts, and as the obesity epidemic continues to soar, more patients with SVI/B who are obese can be expected to be hospitalized.

A 2013 study that secured data from a regional registry in Western Australia discovered that legally blind hospitalized adults had a seven times higher mortality rate compared to those with normal vision [20]. In a longitudinal study from 2002 to 2013 using the Korean National Health Insurance database, Choi also found that those with blindness (> 1200 individuals) also had a higher mortality than patients with normal sight [21]. These results held in distinct analyses assessing both older (> 60 years of age) and younger (< 60 years) patients [21] suggesting that the associations were independent of age. While Crewe or Choi investigated hospitalized patients with blindness, neither study included hospitalized patients with severe vision impairment as was done in our study. In 2013, the World Health Organization (WHO) launched a global action plan for universal eye health with specific guidance for caring for those with SVI/B [22]. These efforts were intended to heighten awareness and escalate the reporting of vision loss in hopes of modifying clinical practice. The current study provides more recent results compared to those from Australia and Korea, while substantiating their findings within a larger cohort. Further, the associations noted among patients hospitalized in the US illustrate that the WHO’s concerns about worse healthcare outcomes among those who are blind are still justified. Though the specific reasons for the higher mortality among SVI/B patients cannot be determined in our observational study, one possible explanation might be that they are presenting to hospital later in the course of illness with more advanced disease.

To explore in-hospital resource utilization, Morse studied two claims databases - Medicare database and Clinformatics DataMart; their objective was to compare the care of older hospitalized patients with and without vision loss [6]. The study found that patients with severe vision loss had longer LOS, more readmissions, and higher hospital costs compared to patients without vision loss. Though our study also found that patients with SVI/B had longer LOS, there were not significantly higher hospital charges compared to those without this disability. The differences in the results might be explained by the fact that our patient population was broader, including younger hospitalized adults. Also, it is possible that the longer LOS may be attributable to a greater level of complexity in coordinating safe post-discharge care for patients with SVI/B. Lack of variance in charges accrued over the protracted time span, and this may be linked to Taheri’s observations that LOS attributable to the last portion of the hospitalization does not significantly contribute to hospital costs [23]. For these very reasons, LOS is not always correlated with hospital costs [24]. Given that a significantly higher number of SVI/B patients were discharge to facilities rather than to their homes, it may be reasonable to presume that they did not amass high charges while awaiting placement. Though we cannot be certain why SVI/B patients were less often discharged home after hospital discharge, it is not unreasonable to speculate that difficulty complying with post-discharge plans and therapies, either real or imagined by the inpatient care team, may have contributed to the decision. Continuation of some therapies after discharge (particularly those involving injections or infusions) may be especially difficult among those with SVI/B; places with some supervision (e.g. rehabilitation, nursing home…) may have been deemed to be safer and associated with a lower risk of readmission than going home – especially for those living alone or without reliable caregivers [25]. While homecare services can be excellent, patients with vision impairments or other disabilities may need more support after discharge necessitating some time in subacute facilities before transitioning back to their homes.

The cohort of hospitalized SVI/B patients who were obese had significantly higher average charges compared to SVI/B patients without obesity; this result is similar to other studies that have examined the impact of obesity among those who are hospitalized [26–28]. However, in contrast to Zizza, our study did not find longer lengths of stay in patients with obesity compared to those without obesity [29]. The prevalence of obesity may be higher among visually impaired people compared to those without vision impairment, and populations with other disabilities [30, 31]. The reasons for this may be related to both challenges with exercising or burning calories, and barriers with securing or preparing a healthful diet. These circumstances may result in hospital-based providers caring for increasing proportions of SVI/B patients who are also obese. Indeed, obesity has been implicated as a risk factor for macular degeneration, glaucoma, cataracts, and diabetic retinopathy, all which may ultimately result in SVI/B [32]. There are ongoing efforts trying to routinize nutritional counseling for all obese patients while they are hospitalized [33].

Several limitations of this study should be considered. First, the NIS is an administrative database wherein data is highly dependent on coding imputations. It is possible that under-coding for SVI/B and obesity may have occurred. Second, the NIS lacks detailed information about visual testing results, lab data or imaging reports, and medications. Thus, an in-depth investigation into the details of our findings was not feasible. Third, special circumstances that might have influenced diagnostic or treatment decisions, such as social factors and patients’ preferences, cannot be determined from administrative databases. Fourth, disease severity and measurements documenting clinical status (improvement/worsening) over the course of the hospitalization are not captured in NIS. Lastly, in observational studies there may be unmeasured and unknown confounders that influence outcomes. Observed associations suggest relationships between variables but do not prove causality.

## Conclusions

Patients with severe vision impairment or blindness have worse clinical outcomes and higher resources utilization when hospitalized compared to those without this disability. Hospital-based healthcare providers should recognize this vulnerability and consider how to optimally care for and serve this group of patients.

## Supplementary information

**Additional file 1**

## Data Availability

Researchers should readily be able to purchase the same databases we did to conduct research here: https://www.distributor.hcup-us.ahrq.gov/Databases.aspx. The authors did not have special access privileges to the NIS databases. Contact information for further guidance on purchase and download at vog.qrha@rotubirtsiDPUCH.

## Abbreviations

SVI/B: Severe Vision Impairment/Blindness.
NIS: National Inpatient Sample.
SEM: Standard Estimated Mean.
aOR: adjusted Odds Ratio.
aMD: adjusted Mean Difference.
CI: Confidence Interval.
ICD-10-CM: International Classification of Diseases, 10th Revision, Clinical Modification.
US: United States.
TX: Texas.
LOS: Length of Stay.
WHO: World Health Organization.
