# Supplementary material for: Severe vision impairment and blindness in hospitalized patients: a retrospective nationwide study
Source: BMC Ophthalmol. 2021 Jun 22;21:263. doi: 10.1186/s12886-021-02021-2 (PMC8220710; doi:10.1186/s12886-021-02021-2)
Supplement: Supplementary file 1 — Additional file 1 [file 12886_2021_2021_MOESM1_ESM.docx]

Supplementary Table A. ICD-10 codes on severe vision loss/blindness and obesity

| ICD-10 code | Clinical Diagnosis |
| --- | --- |
| H54.0X33 | Blindness right eye category 3, blindness left eye category 3 |
| H54.0X34 | Blindness right eye category 3, blindness left eye category 4 |
| H54.0X35 | Blindness right eye category 3, blindness left eye category 5 |
| H54.0X43 | Blindness right eye category 4, blindness left eye category 3 |
| H54.0X44 | Blindness right eye category 4, blindness left eye category 4 |
| H54.0X45 | Blindness right eye category 4, blindness left eye category 5 |
| H54.0X53 | Blindness right eye category 5, blindness left eye category 3 |
| H54.0X54 | Blindness right eye category 5, blindness left eye category 4 |
| H54.0X55 | Blindness right eye category 5, blindness left eye category 5 |
| H54.1132 | Blindness right eye category 3, low vision left eye category 2 |
| H54.1142 | Blindness right eye category 4, low vision left eye category 2 |
| H54.1152 | Blindness right eye category 5, low vision left eye category 2 |
| H54.1223 | Low vision right eye category 2, blindness left eye category 3 |
| H54.1224 | Low vision right eye category 2, blindness left eye category 4 |
| H54.1225 | Low vision right eye category 2, blindness left eye category 5 |
| H54.2X22 | Low vision right eye category 2, low vision left eye category 2 |
| E66.0 | Obesity due to excess calories |
| E66.01 | Morbid-severe obesity |
| E66.09 | Other obesity due to excess calories |
| E66.1 | Drug induced obesity |
| E66.2 | Morbid-severe obesity with alveolar hypoventilation |
| E66.9 | Unspecified obesity |
| Z68.3 | BMI 30-39 |
| Z68.30 | BMI 30.0-30.9 |
| Z68.31 | BMI 31.0-31.9 |
| Z68.32 | BMI 32.0-32.9 |
| Z68.33 | BMI 33.0-33.9 |
| Z68.34 | BMI 34.0-34.9 |
